# Quantification of the flexural rigidity of endovascular surgical devices using three-point bending tests

**DOI:** 10.21203/rs.3.rs-3736325/v1

**Published:** 2023-12-14

**Authors:** Michael Y. Qiu, Charles B. Suskin, Juan J. Becerra-Garcia, Sophia H. Roberts, DeVaughn G. Rucker, Mohamed A. Zayed, Joshua W. Osbun, Guy M. Genin

**Affiliations:** 1 CardioVascular Research Innovation in Surgery & Engineering Center, Washington University in St. Louis, Missouri, USA; 2 Division of Neurotechnology, Department of Neurological Surgery, Washington University School of Medicine, St. Louis, Missouri, USA; 3 Department of Biomedical Engineering, Washington University in St. Louis, St. Louis, Missouri, USA; 4 Department of Mechanical Engineering and Material Sciences, Washington University in St. Louis, St. Louis, Missouri, USA; 5 Center for Innovation in Neuroscience and Technology, Washington University School of Medicine, St. Louis, Missouri, USA; 6 Section of Vascular Surgery, Department of Surgery, Washington University School of Medicine, St. Louis, Missouri, USA; 7 NSF Science and Technology Center for Engineering Mechanobiology, Washington University in St. Louis, St. Louis, Missouri, USA

## Abstract

Endovascular surgical procedures require the navigation of catheters and wires through the vasculature to reach distal target sites. Quantitative frameworks for device selection hold the potential to improve the tracking of endovascular devices through vascular anatomy by personalizing the device flexural rigidity to an individual’s anatomy. However, data are lacking to facilitate this technology, in part because typical endovascular devices have intricate spatial variations in mechanical properties that are challenging and tedious to quantify repeatably. We therefore developed a three-point bend test methodology using a custom rig and applied it to measure lengthwise flexural rigidity profiles of endovascular devices that are used to target the cerebral vasculature. The methodology demonstrated high repeatability and was able to characterize transition zones. We applied the methodology to generate the first comprehensive, quantitative library of device flexural rigidities, spanning guidewires, intermediate guides, and long sheaths. We observed that these three classes of device have properties that fall into distinct ranges. Additional plots examining relationships between flexural rigidity, device diameter, and length revealed application-specific trends in flexural properties. This methodology and the data allow for standardized characterization and comparisons to aid device selection, and have the potential to both enhance surgical planning and inform future innovation.

## Introduction

**Figure 1.**
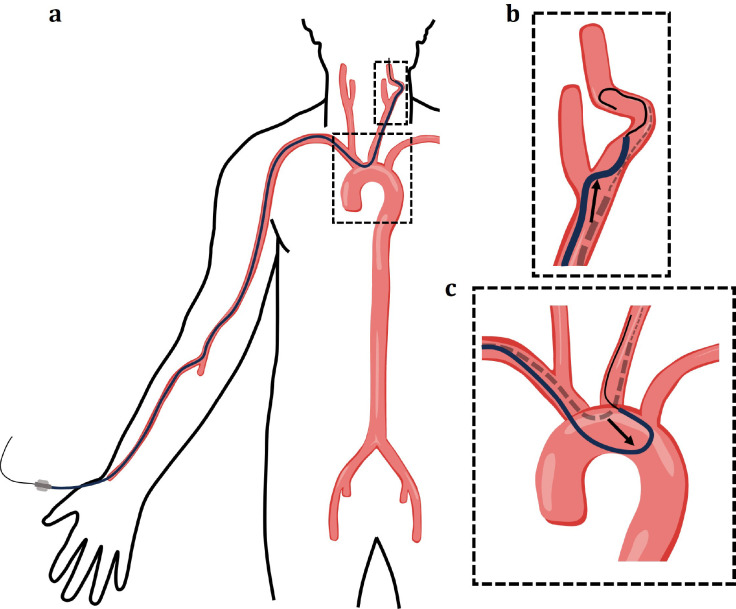
Schematics of anatomical challenges encountered by In endovascular interventionalists while navigating from the access site to the target vessel. (**a**) Diagram depicting the route of navigation for a neuroendovascular procedure using trans-radial access. The endovascular surgical device system is inserted via the radial artery in the right arm and then tracked into the aortic arch before making a sharp turn up the extracranial arteries to access vessels in the brain. (**b)** One navigational challenge often encountered involves vessel tortuosity. Due to twists and turns in a vessels, devices can fail to track and instead kink, loop, or back out of the selected vessel. (**c**) Herniation often occurs at the sharp bend required to navigate up the extracranial vessels via the aortic arch. As depicted, if the supporting device is not stable enough, on advancement of the second device, the entire system will drop and squeeze down the ascending or descending aorta.

Endovascular approaches have transformed the treatment of vascular diseases throughout the body, providing minimally invasive alternatives to more complex open procedures.^1–10^ Endovascular procedures rely on a system of devices consisting of a combination of long hollow tube devices inserted into an artery or vein over a guidewire, and navigated to distal point in a vascular structure of interest. The hollow tube devices can serve as catheters, intermediate guide catheters, or long sheaths, depending on their diameter and intended functionality. For any given endovascular surgical procedure, interventionalists often choose from a variety of devices within each of these categories, with each device having its own performance characteristics. These performance characteristics depend in part upon the mechanical properties of the catheter.^11^ In particular, devices have unique flexural rigidities, or resistance to bending, and often the flexural rigidities will vary along their lengths.^12^ The number, nature, and locations of the transitions in flexural rigidity also vary considerably by device.^13^ The mechanical properties of catheters and wires, especially their flexural rigidity, greatly impact their clinical usability.^11^ In particular, to navigate to a diseased vessel, interventionalists often have to cross multiple anatomical challenges.^14^ For example when treating a stroke using endovascular transradial access (TRA), the devices are inserted via the radial artery in the upper extremity and navigated to the cerebral vessels via the aortic arch in the chest (Figure 1a).^15–18^ Two anatomical challenges are often encountered along this route. First, tortuosity in the carotid arteries can impede the advancement of devices, especially if they are too stiff (Figure 1b).^19–21^ Second, device flexural rigidity has recently been shown to play a driving role in a phenomenon called herniation, which can occur during device navigation across sharp bends or turns in the vasculature; in this case when navigating down into the aortic arch and then turning sharply upwards to the carotid artery.^11^ Herniation occurs when the supporting guide wire or catheter used to select the carotid is unable to support the advancement of a telescoping device such as another catheter, intermediate guide catheter, or sheath. This results in the entire system dropping down and herniating out of the carotid artery and into the aortic arch, which prevents further advancement and delays the necessary treatment (Figure 1c).^11^

Interventionalists often must balance both navigating tortuosity and avoiding herniation when making device selections.^22^ However, those needs can be in conflict. Less stiff device combinations offer improved ability to navigate tortuosity, but may fail to support therapeutic device delivery, like stents and coils. On the other hand, stiffer devices offer more support to avoid herniation, but have a more difficult time crossing tortuosity. Furthermore, device selection will change significantly depending on target vessel and patient specific anatomy.^22^ Complicating things further is the fact that device manufacturers rarely publish quantitative data on the mechanical properties of their endovascular devices. Interventionalists, therefore, rely primarily on prior experience, practice patterns, and subjective qualitative metrics (“stiffer” vs. more “floppy”) to make decisions on device selection for a particular surgery.^23–25^ This is both imprecise and a skill that often requires years to develop.^22^

Thus, there is a pressing need for the quantitative characterization of the flexural rigidity of endovascular devices. However, no standard protocols for the measurement of device flexural rigidity currently exist. Current mechanical test instruments are often not well suited to the task, with inappropriate force measurement ranges and test fixtures not designed to accommodate long endovascular devices. Other past efforts to characterize endovascular devices have utilized a cantilevered beam method.^12,13^ This, however, has limitations in that it requires the devices to be cut into sections and can only be used to characterize homogeneous regions. Thus, it cannot characterize transition zones or regions of variable flexural rigidity.

Here, we solve the problem with a novel test rig and testing methodology, based on the well-established three-point bend test,^26^ that is capable of quantitatively and consistently characterizing the flexural rigidities of endovascular surgical devices along their entire length, including transition zones. Using this methodology, we then characterize the most commonly used neuroendovascular surgical devices, generating the first quantitative library of device flexural rigidity profiles.

## Methods

### Device Construction and Design

#### CNC Frame

A 3-axis CNC router (Genmitsu 3018-PROVer) was used for the base of the three-point bend device. The CNC gantry was modified by removing the spindle motor to accommodate a custom load cell fixture attached via bolts to the Z-axis carriage. A load cell fixture was made of a combination of 3D printed parts, off-the-shelf components, and machined parts. The Z-axis stepper motor was used to advance and retract the loading pin by raising and lowering the Z-axis carriage.

#### Custom Three-Point Bend Test Fixture

A custom three-point bend test fixture was made that attaches to the CNC frame. Two support pins were secured to 3D printed fixtures that bolt down to the bed of the CNC. Additional rollers were attached to the 3D printed fixtures above and slightly offset of the two support pins to assist in aligning the test specimen without affecting the load measurement. Two extruded aluminum rails were then bolted to the bed, one on either side of the two support pins. The inner surface of the extruded rail was lined up with the top of the support pins to allow for a single uniform surface to support the full length of the test specimen. The full test fixture was made and assembled using a combination of 3D printed parts, off-the-shelf components, and machined parts.

#### Load Cell and Control Electronics

The signal from the 500g load cell (Sparkfun TAL221) was amplified using a load cell amplifier (Sparkfun HX711) before being fed into a microcontroller (Arduino Uno Rev3). The load cell was calibrated using the standard method with a series of test weights. Control of the CNC gantry was accomplished using the factory GRBL motor control board included with the 3018-PROVer.

#### Control Software

Communication with the device was established over two USB connections: one to the Arduino microcontroller for data acquisition and one to the motor control board for positioning of the loading pin. Motor control was done using G-code scripts executed in the GRBL controller software Candle. Data acquisition was done using PuTTY.

### Measurement Protocol

For each device, starting from the distal end, marks were made at 1 cm increments through the distal transition zones to indicate each test point location. At the main shaft, 5 cm increments were used given that the main shaft has a constant construction. Test points were marked up to 40–55 cm from the distal tip depending on the device and extent of the transition zones. For each test, a test point was centered under the loading pin, which was then lowered at a constant feed rate of 0.5 mm/s for 2 mm. The loading pin was held for 5 seconds, before being retracted. After one test point was measured, the device was manually advanced to the next test point. The force applied by the loading pin as measured by the load cell was recorded continuously using the Arduino microcontroller. For all measurements, the distance between the support pins was set at 30 mm.

### Calculation of Flexural Rigidity

Following acquisition of the load cell measurements, the raw force data was used to calculate flexural rigidity using a custom script written in MATLAB. Time stamps for each force measurement were converted to displacements using the known feed rate specified in Candle. The zero of displacement was taken to be the time stamp where the force first increases during each test. From the force-displacement plot, the flexural rigidity was then calculated by finding the slope of the rise in the force peak associated with each test and then plugging that slope into the three-point bend equation for flexural rigidity.

## Results

### An Apparatus to Quantify Spatial Variations of Device Flexural Rigidity

**Figure 2.**
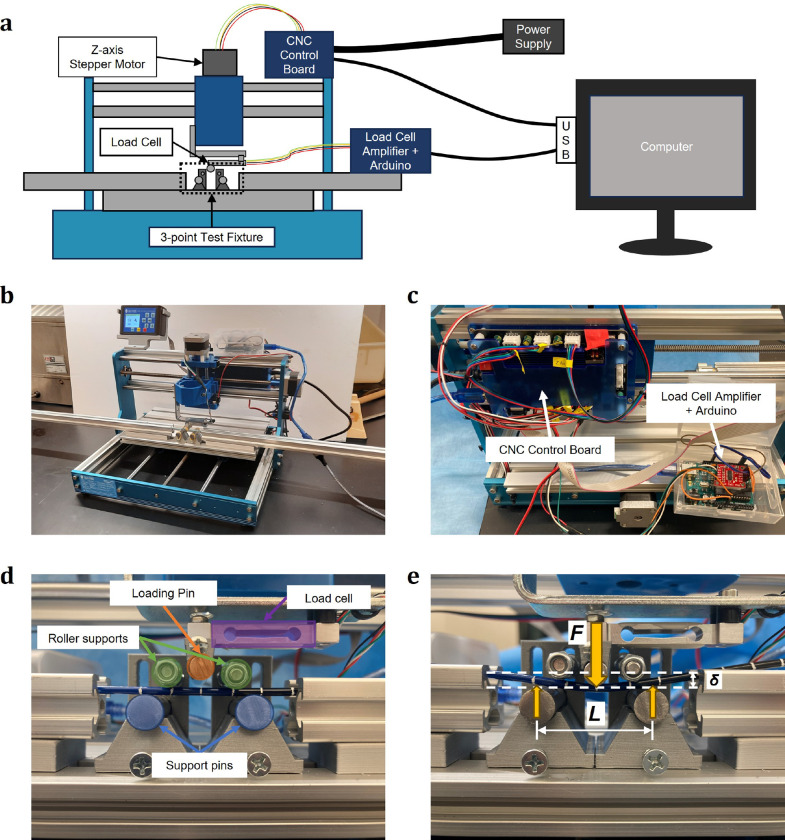
Three-point bend device. (**a**) Device schematic illustrating electrical components and connections. (**b**) Image of the overall device. (**c**) The primary electronics include an off-the-shelf CNC control board and a load cell data acquisition circuit based on an Arduino microcontroller. (**d**) A custom three-point bend fixture was designed and integrated into the CNC base platform. The loading pin attaches to the load cell, which is fixed to the Z-axis carriage. Pins beneath the test specimen support it from below, while roller pins above help align the specimen without affecting the load measurement. (**e**) Diagram showing the key parameters measured in the three-point bend test.

To reliably and accurately characterize flexural rigidity along the entire length of a catheter, we designed and built a custom a three-point bend test rig (Figure 2a). The test rig utilized an off-the-shelf 3-axis computer numerical control (CNC) router as its base (Figure 2b). We modified the CNC by removing the spindle motor and mounting a custom load cell fixture to the Z-axis carriage, allowing the raising and lowering of the loading pin using the Z-axis motor. Control of the Z-axis motor was achieved using G-code scripts sent to the CNC control board included with the CNC router, while data acquisition from the load cell was performed by a load cell amplifier connected to an Arduino microcontroller (Figure 2c). We attached a custom three-point bend fixture to the CNC bed designed specifically to support testing of long endovascular surgical devices (Figure 2d). This fixture included two support pins below the device that served as two of the points in the three-point bend test. Additional roller supports above the device and aluminum channel on either side of the support pins held and aligned the device during testing.

Using this set-up, we calculated flexural rigidity directly from the force measured by the load cell as the loading pin was lowered onto the device (Figure 2e) by modeling the device as an Euler-Bernoulli beam. For a linear elastic Euler-Bernoulli beam, the flexural rigidity, EI, could be written as:

(1) $$EI=\frac{FL^{3}}{48\delta }$$

where δ is the maximum deflection at the center of the section of beam between the two support pins, F is the force needed to cause a deflection of δ, and L is the distance between the support pins (Figure 2e).^27^ In our experiments, multiple data points during loading of the catheter were analyzed, and the slope m=F/δ was estimated in the least squares sense, such that:

(2) $$EI=m\frac{L^{3}}{48}$$

**Figure 3.**
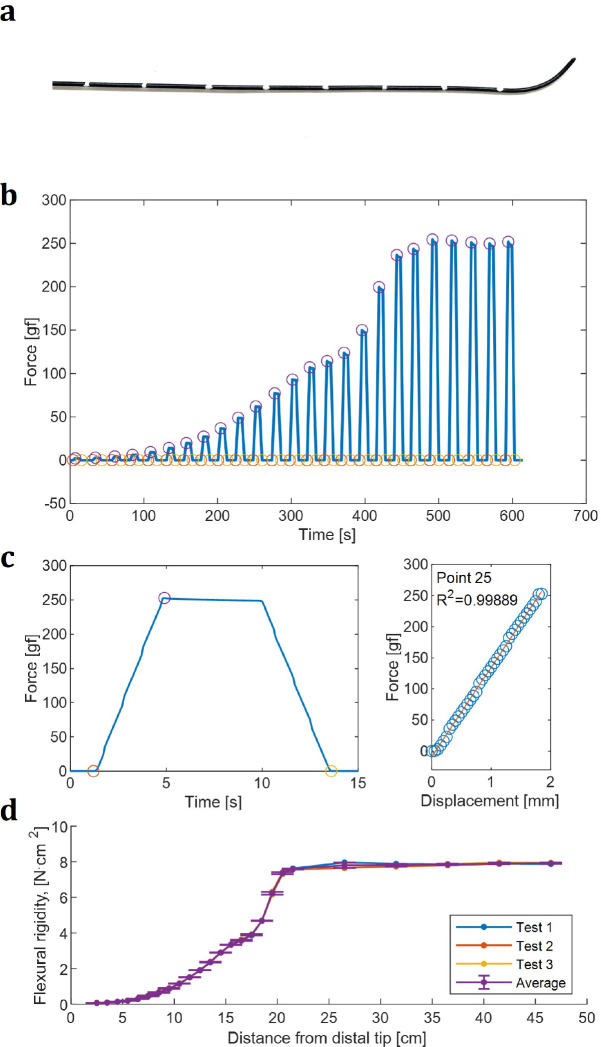
Data acquisition and flexural rigidity calculation. (**a**) Starting from the distal end, test points are marked on each device at 1 cm increments through transition zones followed by 5 cm increments after reaching the main shaft. These markings are then used to center the test point under the loading pin. Example image shows the distal end of an 0.035” Stiff Roadrunner guidewire, and subsequent plots correspond to this wire. (**b**) At each test point, the force measured by the load cell was recorded as the loading pin is lowered. After each test, the device was manually advanced to the next test point, while recording continuously. After all points were tested, a custom MATLAB script was used to identify the start, peak, and end of each test - indicated by the orange, purple, and yellow circles, respectively. (**c**) Using the same MATLAB script, the linear rise of each force peak was extracted and the time was converted to displacement using the specified feed rate. A linear regression was performed to calculate the slope, which was then used to calculate flexural rigidity. Example shown is for the 25th peak in (**b**), corresponding to the 25th test point. (**d**) A subsequent plot generated of the lengthwise flexural rigidity of the 0.035” Stiff Roadrunner. All tests were repeated three times, with results demonstrating the high precision and repeatability of the testing methodology.

The test was repeated at multiple test points along the length of a device (Figure 3a) as it was drawn down a guide track to quantify the flexural rigidity variations along the device’s length. Force data acquired continuously from the load cell during indentation at each point, using a set loading rate and maximum displacement, were recorded and processed using linear regression to estimate m (Figure 3b,c), which was used with Equation 2 to estimate EI. At higher loads, viscoleastic relaxation of a few percent was observed (Figure 3b), indicating that the values of m were likely elevated by a few percent due to rate-dependent effects.

Individual flexural rigidity measurements were compiled to quantify the full flexural rigidity profile for a device (Figure 3d).

### Device Flexural Rigidity Measurements

We used the three-point bend test rig to quantify the flexural rigidity profiles of a library of commonly used neuroendovascular devices. This library consisted of 9 guidewires, 5 intermediate guide catheters, and 5 long sheaths. Each device was tested three times. See Table 1 for full list of devices tested.

**Figure 4.**
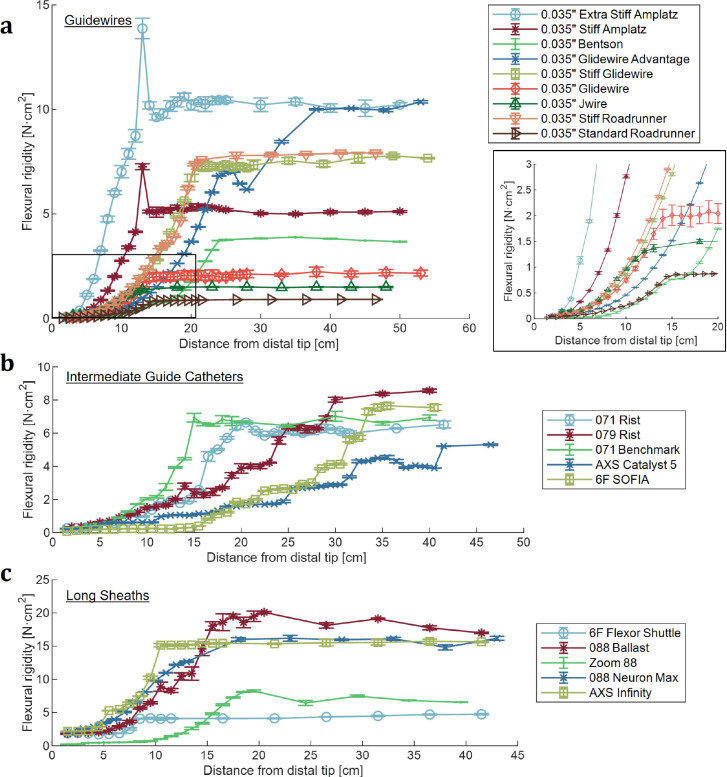
Endovascular surgical devices have flexural rigidities that vary significantly along their length with each device having its own unique flexural rigidity profile. (**a**) Flexural rigidity profiles of guidewires. Inset shows a zoom-in of the varying distal region. The transition from the distal region to the main shaft occurs in different locations ranging from 15–30cm from the tip depending on guidewire. (**b**) Flexural rigidity profiles of intermediate guide catheters. Intermediate guide catheters have multiple transition zones with the number and location of transitions differing between devices. (**c**) Flexural rigidity profiles of long sheaths.

The guidewires typically possessed a flexible distal tip region that transitioned to a stiffer main shaft (Figure 4a). Main shaft stiffness varied considerably, spanning a range of flexural rigidities from ~1–13 N·cm^2^. The length of the distal transition region varied between guidewires, with the transition to the main shaft occurring 15–25 cm from the distal tip depending on guidewire. The nature of the transition also differed, with some wires showing a more gradual increase in stiffness, while showing a higher stiffness gradient. Two guidewires, the 0.035” Stiff Amplatz and 0.035” Extra Stiff Amplatz, showed a spike in stiffness at the transition to the main shaft corresponding to the location of a metal weld.

The intermediate guide catheters, unlike the guidewires, possessed multiple distinct stiffness transition regions (Figure 4b). Again, the location and slope of these transitions varied considerably between devices. The 6F SOFIA, in particular, possessed a much more flexible distal tip that extended for longer than any of the other intermediate guides. Main shaft stiffness for intermediate guides ranged from ~5–10 N·cm^2^.

Similarly, the long sheaths each had unique profiles (Figure 4c). The Zoom 88 differed from other long sheaths by having a 20 cm extended distal tip region with distinctly lower flexural rigidity. Main shaft stiffness for the long sheaths ranged from ~5–25 N·cm^2^, with three of the devices (the 088 Ballast, AXS Infinity, and 088 Neuron Max) clustered at the high end of the range and the other two (the 6F Flexor Shuttle and Zoom 88) at the lower end.

### Device Categorization by Flexural Rigidity

**Figure 5.**
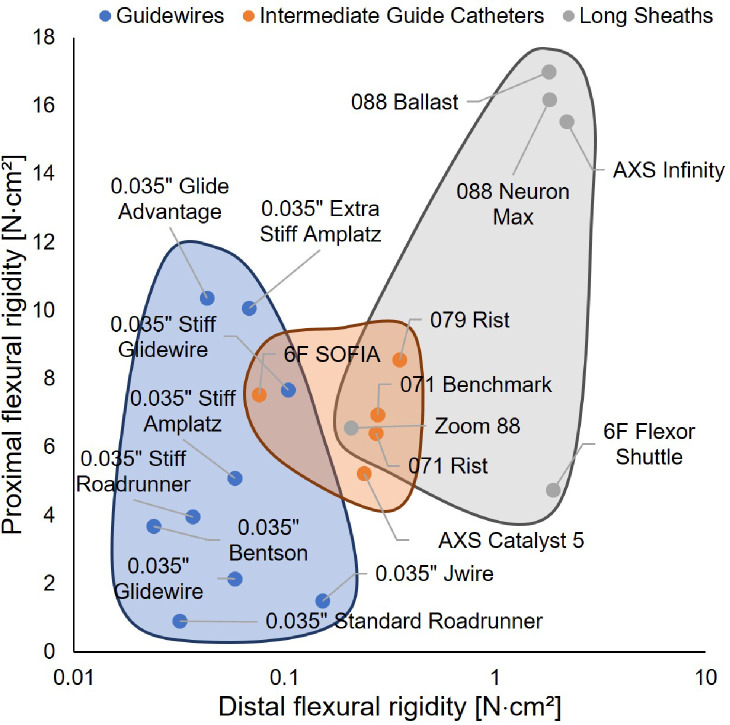
Traditional classifications for devices generally translate into distinct flexural rigidity groupings. Ashby plot showing distal-proximal flexural rigidity relationships for the different devices colored by classification. Devices of each classification occupy a distinct region of the plot. The 6F SOFIA and Zoom 88 are outliers that cross the boundaries of traditional device classifications with distinctly lower distal flexural rigidities. Note that the x-axis is log-scaled.

We plotted these data for the 19 devices tested on Ashby plots to study functional similarities and differences between devices based on their flexural rigidity. Values used to generate the Ashby plots can be found in Table 1. First, we examined the relationship between distal and proximal flexural rigidity (Figure 5). We found that devices generally clustered into distinct groupings that correspond to their traditional classifications. The guidewires grouped together on the lower end of the distal flexural rigidity range. The intermediate guide catheters occupied a smaller region in the intermediate range of both proximal and distal flexural rigidity. The long sheaths occupied a region at the higher end of the distal flexural rigidity range, with proximal flexural rigidities ranging from the middle to the extreme high end of the range. Notably, two devices stood out as “hybrids”: the 6 F SOFIA and the Zoom 88. These devices crossed the boundaries of traditional device classifications with distinctly lower distal flexural rigidities than other intermediate guide catheters or long sheaths.

**Figure 6.**
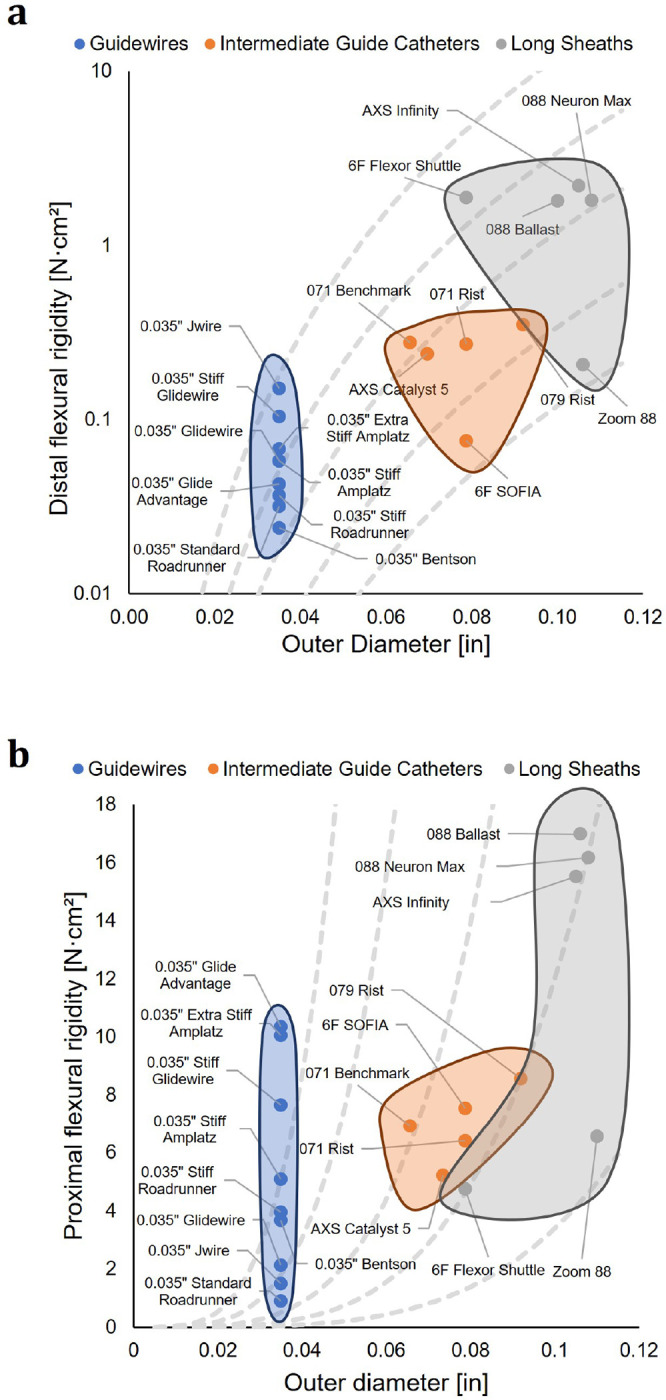
Device outer diameter corresponds with traditional classification as well as distal and proximal flexural rigidity. (**a**) Ashby plot showing the relationship between device outer diameter and distal flexural rigidity for the different device classifications. Long sheaths tend to be the largest devices, followed by intermediate guide catheters, and then guidewires. Distal flexural rigidity generally increases with device outer diameter. Note that the y-axis is log-scaled. Grey dashed lines represent isoclines of constant Young’s modulus and self-similar catheter cross-section. (**b**) Ashby plot showing the relationship between device outer diameter and proximal flexural rigidity for the different device classifications. Guidewires span nearly the full range of proximal flexural rigidities. Intermediate guides catheters occupy the middle of the range, while long sheaths occupy the middle to extreme high end. Grey dashed lines represent isoclines of constant Young’s modulus and self-similar catheter cross-section.

We next generated Ashby plots displaying relationships between distal flexural rigidity and device outer diameter (Figure 6a) and proximal flexural rigidity and device outer diameter (Figure 6b). Guidewires had the smallest outer diameters, followed by intermediate guide catheters, and then long sheaths, which corresponds to their placement in a coaxial system. Generally, distal flexural rigidity increased with increasing outer diameter (Figure 6a). Proximal flexural rigidity appears to have less of a direct relationship with outer diameter, and instead likely depends on device design and construction (Figure 6b). The 0.035” Stiff Glidewire, for example, is designed and constructed to have a stiffer main shaft than its normal 0.035” Glidewire counterpart.

## Discussion

The testing methodology for quantifying the lengthwise flexural rigidity of endovascular surgical devices was applied to compile the first publicly-available, comprehensive library of lengthwise flexural rigidity profiles for commercially available endovascular surgical devices used for neurovascular procedures such as stroke. The test rig was assembled using off-the shelf parts, making it both relatively inexpensive and intuitive to use, and has the additional advantage that it can accommodate testing of long specimens. Other methodologies require either cutting the device into short segments^12,13^ or limiting testing to small segments of a catheter.^26^ These destructive or spatially limited approaches are especially inconvenient for working with novel, one-off prototypes. By incorporating a rail into the three-point bend fixture, our test rig enables long specimens to be tested non-destructively, and measurements can be taken quickly at any point along their lengths. The test rig utilizes a CNC base and is controlled by G-code, which is industry standard in manufacturing and precision machining, is compatible with a vast library of automation scripts and control programs, and can be adapted to accommodate additional testing procedures such as cyclic testing or custom load-unload profiles.

Certain limitations with this testing method need to be noted. The linear, Euler-Bernoulli beam theory applied assumes that the geometry of the cross-sections of the beam do not change in a significant manner under load and remain planar to the neutral axis. This is largely valid for the small deflections (< 2 mm) and loads used in our testing,^26^ but would be inaccurate at larger deflections. The theory used to interpret data assumes zero moment at the free ends, and in cases of large overhang by segments of weight that is comparable to the indentation force, vertical restraint of the overhanging catheter might be warranted, and the formula for indentation of a clamped beam might need to be used. Finally, in this study we quantified only the instantaneous flexural rigidity. Viscoelastic effects, in which isometric forces can change in a time dependent manner, were on the order of a few percent for the catheters studied, which is small compared to measurement repeatability. Exploration of strain or time-dependent mechanical properties can be evaluated using the test rig developed here, and is an avenue for further study.

The data acquired in this study demonstrated that endovascular surgical devices possess widely varying flexural rigidity properties, including differences in the number and nature of their transition zones and the stiffness of their main shafts. Generally, distal flexural rigidity increased with increasing outer diameter (Figure 6a). For both distal and proximal flexural rigidity, intermediate guide catheters and sheaths tracked along the same range of scale lines (gray, dashed lines in Figure 6a–b), which are isoclines for self-similar catheters with identical material properties but different outer diameter. These lines scale as the outer diameter raised to the fourth power, and reveal the impact of differences in catheter thickness and selection. Guidewires are often made of metal wires, and lie on isoclines far above both distal and proximal stiffnesses of intermediate guide catheters and sheaths.

Understanding the flexural rigidity profiles of endovascular devices is paramount for successful clinical management of neurovascular pathologies such as stroke. During these procedures, time is often of the essence in order to recover appropriate blood flow to the brain penumbra. Extended endovascular navigation, or repeated challenges in gaining stable access to the distal neurovascular structure of interest can lead to delay in treatment and lasting consequences to the patient. While interventionalists rely heavily on their past experiences and clinical pattern recognition, patient anatomy is highly variable, and no one patient ever has the same vascular anatomy as another. As such, subjective assessments can sometimes be misleading, and often require ‘on-the-spot,’ creative approaches to treat the patient. Additionally, catheter herniation itself can lead to arterial trauma and dissection. The sudden jolt of force exerted by the catheter as it herniates out of a distal vascular structure can injure the sensitive vascular wall, further complicating the procedure and increasing the risk of negative perioperative consequences. Having objective, quantitative flexural rigidity profiles that interventionalists can reference while making initial device selections can help avoid these problems

Understanding the standard flexural rigidity properties of endovascular devices can also enhance personalization of endovascular procedures for specific patient vascular anatomies. Supporting this concept, we observed that guidewires grouped together on the lower end of the distal flexural rigidity range. Functionally, this is expected because guidewires are typically advanced into the narrow vessels and used to select vessels and cross lesions, meaning that they must have soft flexible tips. The proximal flexural rigidities of the main shafts of guidewires spanned a wide range, giving options for more choices to interventionlists. Such a range of properties is desirable as an interventionlist chooses devices to, for example, cross tortuous vasculature or provide more stiffness for added support. Intermediate guide catheters attempt to balance support and flexibility, and occupied the middle range of the Ashby plots (Figure 5). Most sheaths were substantially stiffer than guidewires and intermediate guide catheters at their distal ends, with proximal flexural rigidities ranging from the middle to the extreme high end of the range. This corresponds functionally as long sheaths prioritize support over flexibility.

Our study findings offer a comprehensive, quantitative assessment of the endovascular devices available, which may help with more informed, consistent, and optimized device selection during neurovascular procedures. These quantitative data could also potentially be used to inform future research and innovation. In particular, the Ashby plots highlight openings in the design space that could be filled by new devices, giving interventionalists more options. Quantitative flexural rigidity data are also essential for the development of physics models aiming to explain the mechanics of endovascular device navigation as well as algorithms underlying robotic endovascular surgery platforms. These data, combined with models for navigation, can contribute to future automated device selection based on patient-specific anatomy.

## Figures and Tables

**Table 1.** Distal and proximal flexural rigidities along with outer diameters of all devices tested. The distal value was taken from the test point closest to the distal tip. The proximal values were taken from a test point on the main shaft approximately 40–55 cm from the distal tip depending on device. Outer diameters were referenced from manufacturer’s specifications. Data are presented as mean ± standard deviation.

	Distal Flexural	Proximal Flexural	Distal	Proximal
	Rigidity [N cm^2^]	Rigidity [N cm^2^]	OD [in]	OD [in]

**Guidewires**				

0.035” Glidewire	0.06 ± 0.001	2.13 ± 0.142	0.035	0.035
0.035” Stiff Glidewire	0.10 ± 0.001	7.67 ± 0.029	0.035	0.035
0.035” Glide Advantage	0.04 ± 0.003	10.4 ± 0.056	0.035	0.035
0.035” Stiff Amplatz	0.06 ± 0.009	5.09 ± 0.045	0.035	0.035
0.035” Extra Stiff Amplatz	0.07 ± 0.012	10.1 ± 0.088	0.035	0.035
0.035” Standard Roadrunner	0.03 ± 0.003	0.91 ± 0.003	0.035	0.035
0.035” Stiff Roadrunner	0.04 ± 0.001	3.96 ± 0.018	0.035	0.035
0.035” Bentson	0.02 ± 0.007	3.68 ± 0.015	0.035	0.035
0.035” Jwire	0.15 ± 0.027	1.50 ± 0.031	0.035	0.035

**Intermediate Guide Catheters**				

071 Rist	0.27 ± 0.005	6.41 ± 0.210	0.079	0.079
079 Rist	0.35 ± 0.010	8.57 ± 0.099	0.092	0.092
071 Benchmark	0.28 ± 0.011	6.94 ± 0.158	0.066	0.066
AXS Catalyst 5	0.24 ± 0.008	5.22 ± 0.018	0.070	0.074
6F SOFIA	0.08 ± 0.003	7.54 ± 0.174	0.079	0.079

**Long Sheaths**				

Zoom 88	0.21 ± 0.014	6.57 ± 0.039	0.106	0.110
AXS Infinity	2.20 ± 0.013	15.5 ± 0.117	0.105	0.105
088 Neuron Max	1.82 ± 0.187	16.2 ± 0.295	0.108	0.108
088 Ballast	1.81 ± 0.061	17.0 ± 0.091	0.100	0.106
6F Flexor Shuttle	1.88 ± 0.181	4.75 ± 0.109	0.079	0.079
